# *Hd1* Allele Types and Their Associations with Major Agronomic Traits in Korean Rice Cultivars

**DOI:** 10.3390/plants10112408

**Published:** 2021-11-08

**Authors:** Youngjun Mo, Chang-Min Lee, Hyang-Mi Park, Su-Kyung Ha, Mi-Jung Kim, Jieun Kwak, Hyun-Sook Lee, Jeong-Heui Lee, Ji-Ung Jeung

**Affiliations:** 1 National Institute of Crop Science, Rural Development Administration, Wanju 55365, Korea; yjmo@jbnu.edu (Y.M.); cropas@korea.kr (C.-M.L.); parkhm2002@korea.kr (H.-M.P.); rocksue193@korea.kr (S.-K.H.); kumozi@korea.kr (M.-J.K.); jieun74@korea.kr (J.K.); leehs0107@korea.kr (H.-S.L.); lejehe@korea.kr (J.-H.L.); 2 Department of Crop Science and Biotechnology, Jeonbuk National University, Jeonju 54896, Korea

**Keywords:** rice, *Hd1*, days to heading, culm length, amylose content, protein content

## Abstract

Optimizing flowering time in crop plants is critical for maximizing yield and quality under target environments. While there is a wide range of heading date variation in Korean rice cultivars, the underlying gene mechanisms are unclear. Here, we sequenced the protein coding regions of *Hd1*, the major rice heading date gene, from 293 Korean rice cultivars and investigated the associations between *Hd1* allele types and major agronomic traits under four different environments. There were four functional *Hd1* and five nonfunctional *hd1* alleles distributed among the 293 Korean rice cultivars. The effects of the *Hd1* allele types were highly significant for days to heading in all four environments, explaining 51.4–65.8% of the phenotypic variation. On average, cultivars carrying nonfunctional *hd1* headed 13.7 days earlier than those carrying functional *Hd1*. While the *Hd1* allele types exhibited highly significant effects on culm length and protein content under all four environments, the differences between cultivars carrying *Hd1* and *hd1* were minimal. The effects of the *Hd1* allele types on amylose content were significant in only one of the four environments. Our results provide useful information for fine-tuning rice heading dates by utilizing different *Hd1* alleles in rice breeding programs.

## 1. Introduction

The transition from the vegetative to reproductive phase is a critical developmental event in plants. To ensure offspring survival under favorable environments, the onset of reproductive development is regulated by intricate genetic mechanisms sensing various endogenous cues and environmental conditions such as daylength and temperature [1,2]. In agriculture, flowering time control is important as it determines the regional adaptability of different crop species and affects a range of agronomic traits relevant to crop yield and quality [3]. In rice, the vegetative-to-reproductive transition is induced under short day condition when the photoperiod becomes shorter than the critical daylength [4]. As photoperiod sensitivity shows wide variation among different rice genotypes, the use of germplasm with varying degrees of photoperiod sensitivity enabled the expansion of rice cultivation from the tropics to high latitude regions [5,6,7,8,9].

The main genetic factors involved in rice flowering time (aka heading date) control are the *Hd3a* and *RFT1* genes, the orthologs of Arabidopsis *Flowering Locus T* (*FT*) [10,11]. These genes are upregulated under short day conditions and produce florigen, the mobile flowering signal that moves from leaves to the shoot apical meristem through the phloem, and induces floral development. The expression level of *Hd3a* shows a strong correlation with the rice heading date, with the rice genotypes exhibiting higher *Hd3a* expression in the leaves heading earlier [12]. While the amino acid sequences of *Hd3a* are highly conserved across diverse rice genotypes, sequence variations in the promoter region of *Hd3a* and those of the upstream genes controlling the *Hd3a* expression largely determine the heading date variation of cultivated rice [12,13].

Among the many rice heading date genes isolated so far [14], *Hd1* exhibits higher sequence polymorphisms than the other genes (e.g., *Ehd1*, *OsMADS51*, *Ghd7*, *DTH8*, *OsPRR37*, *Hd3a*, and *RFT1*) and its allelic variations are strongly associated with the heading date variation of rice [12,13,15]. As *Hd1* possesses a dual function of enhancing and repressing flowering under short days and long days, respectively [16], the loss-of-function *hd1* alleles play an important role in expanding rice adaptability in high latitude areas where the day length is long and winter comes early [6,8]. The nonfunctional *hd1* alleles are also useful for breeding high yielding temperate japonica rice cultivars adapted in the tropics, as the cultivars with functional *Hd1* show an extremely early heading phenotype without sufficient vegetative growth due to the short day length in the tropical regions [15,17].

At least 17 nonfunctional *hd1* alleles have been reported so far [8,12,13,15,16,18]. Initially, premature stop codon mutations were identified in the indica cultivar Kasalath carrying a 2 bp deletion in exon 1 and the japonica mutagenized cultivar HS66 carrying a 43 bp insertion in exon 2 of *Hd1*, and both mutations were reported to enhance heading under natural long day conditions [16]. Subsequently, sequencing analyses of *Hd1* from diverse rice germplasm identified frameshift *hd1* mutations due to small insertions and deletions (Types 2, 3, 7, 13, 14, 15, 16, and 17) and a nonsense mutation (Type 12) [12]. Additional *hd1* alleles have been identified from world-wide rice collections (hap 14, 16, 17, 24, and 29) [13], the International Rice Research Institute (IRRI)’s genebank accessions (*Hd1*-NT1, and Types 20, 21, and 22) [15,18], and rice cultivars adapted in the Hokkaido region of Japan (HW-type, AK-type) [8].

In South Korea, rice cultivars are generally classified into three maturity groups according to heading dates under optimal planting conditions (sowing in early May and transplanting in early June in the southern plain area)—namely, the early maturing group heading before the end of July, the mid maturing group heading in early August, and the mid-late maturing group heading in mid-late August or later [19]. A wide variation exists in the heading dates of Korean rice cultivars, ranging from 46 to 111 days from transplanting to heading under optimal planting conditions [20,21]. However, the genetic basis underlying the heading date variation of Korean rice cultivars have been unclear. In this study, we sequenced the protein coding regions of *Hd1* from 293 Korean rice cultivars released between 1979 and 2017, evaluated the associations between the *Hd1* allele types and the major agronomic traits, and discussed their implications in rice breeding programs.

## 2. Results

### 2.1. Major Agronomic Traits of Korean Rice Cultivars under Different Environments

**Figure 1:**
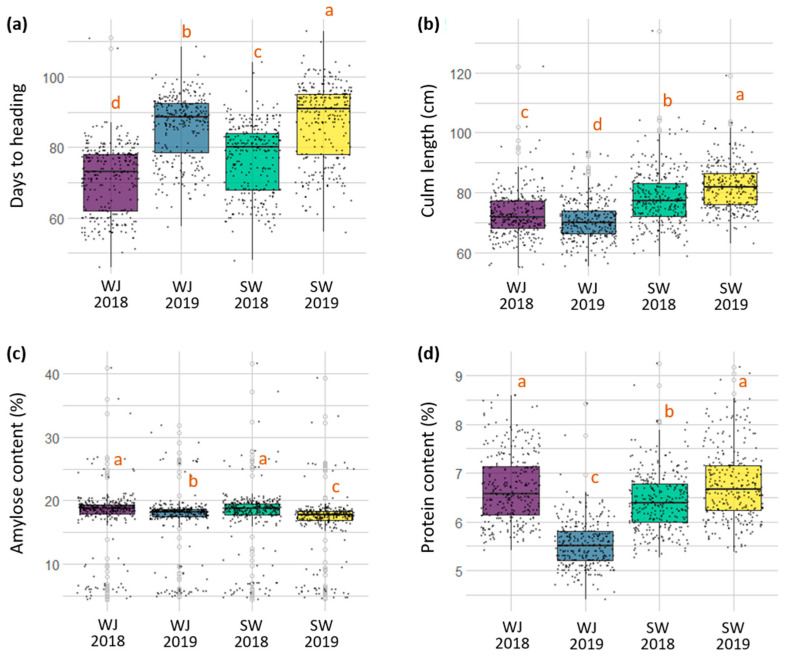
Variation in days to heading (**a**), culm length (**b**), amylose content (**c**), and protein content (**d**) of 293 Korean rice cultivars evaluated at two different locations (WJ—Wanju; SW—Suwon) for two years (2018 and 2019). Different lowercase letters above the box plots indicate a significant difference according to Tukey’s multiple comparisons at *p* < 0.05.

**Figure 2:**
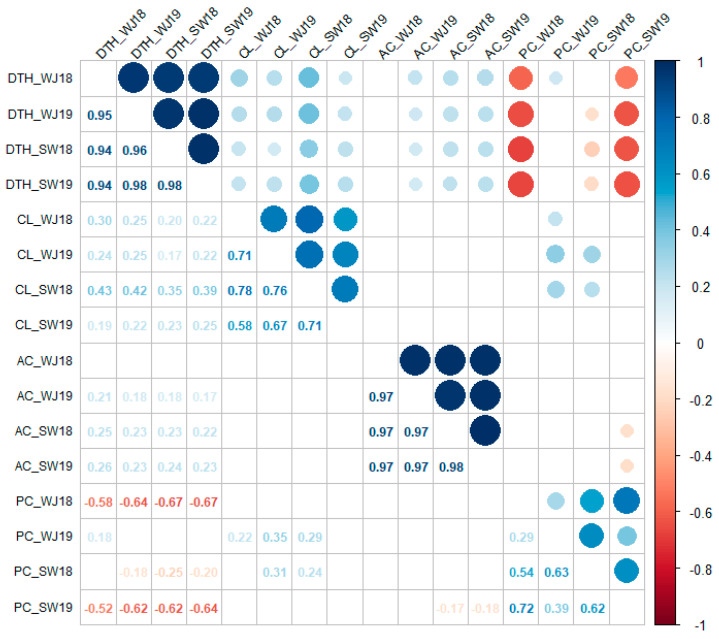
Correlations among days to heading (DTH), culm length (CL), amylose content (AC), and protein content (PC) of 293 Korean elite rice cultivars evaluated at two different locations (WJ—Wanju; SW—Suwon) in two years (2018 and 2019). Pearson’s correlation coefficients significant at *p* < 0.01 are shown in the lower matrix. The size and color thickness of each circle in the upper matrix are proportional to the relevant correlation coefficient.

The days to heading, culm length, amylose content, and protein content of 293 Korean rice cultivars were evaluated at two different field locations (Wanju and Suwon, South Korea) for two years (2018 and 2019; Figure 1 and Figure 2). The plants at both locations were grown under the optimum planting condition in 2018 and early planting condition in 2019 (see Materials and Methods for sowing and transplanting dates).

Days to heading (DTH): In 2018, the average DTH of the 293 cultivars were 71.4 days in Wanju and 77.0 days in Suwon, with a range of 46–111 days and 48–104 days, respectively (Figure 1a). In 2019, the average DTH were 85.8 days (58–109 days) in Wanju and 87.2 days (56–113 days) in Suwon, which were delayed by 14.4 days and 10.2 days, respectively, compared with those at each location in 2018. Despite the large difference in DTH between the optimum planting (2018) and early plating (2019) conditions, the DTH of the 293 cultivars exhibited strong positive correlations (Pearson’s r between 0.94 and 0.98) across four different environments (Figure 2).

Culm length (CL): The average CL of the 293 cultivars was 72.8 cm (55–122 cm) in Wanju and 78.2 cm (59–134 cm) in Suwon in 2018, and 70.8 cm (55–93 cm) in Wanju and 82.1 cm (63–119 cm) in Suwon in 2019 (Figure 1b). While DTH were delayed by early planting (2019) compared with optimum planting (2018) at both locations, the effect of planting time on CL was not consistent between the two locations. The correlations of CL across four different environments were positive (0.58 < r < 0.78), but weaker than those of DTH (0.94 < r < 0.98) (Figure 2).

Amylose content (AC): The average AC of the 293 cultivars was 17.9% (4.5–40.8%) in Wanju and 17.9% (4.5–41.6%) in Suwon in 2018, and 17.3% (4.9–31.8%) in Wanju and 16.9% (4.6–29.2%) in Suwon in 2019 (Figure 1c). Compared with the other traits, the AC in each environment exhibited a greater number of outliers due to the inclusion of waxy rice cultivars (e.g., cultivars with “chal”, meaning “glutinous”, in their names in Table 1) with the AC range of 4.5–7.1% and high amylose cultivars (e.g., cultivars with “goami”, meaning “high amylose”, in their names in Table 1) with the AC range of 25.1–41.6%. Similar to DTH (0.94 < r < 0.98), the AC across four different environments showed very strong positive correlations (0.97 < r < 0.98) (Figure 2), indicating that both DTH and AC are largely determined by genetic factors.

Protein content (PC): The average PC of the 293 cultivars was 6.7% (5.4–8.6%) in Wanju and 6.4% (5.3–9.3%) in Suwon in 2018, and 5.5% (4.4–8.4%) in Wanju and 6.8% (5.4–9.2%) in Suwon in 2019 (Figure 1d). The correlations of PC across four different environments were positive but weaker (0.29 < r < 0.72) than the other traits, i.e., DTH (0.94 < r < 0.98), CL (0.58 < r < 0.78), and AC (0.97 < r < 0.98).

Correlations between different traits: The DTH–CL correlation was significant and weakly positive in all four environments (0.25 < r < 0.35) (Figure 2). The DTH–AC correlation was significant in three of the four environments and was weakly positive (0.18 < r < 0.23). The DTH–PC correlation was significant and negative (−0.64 < r < −0.25) in three of the four environments. There was no significant correlation between CL and AC in all four environments. Significant but weak correlations were observed between CL and PC (0.24 < r < 0.35) in two environments, and between AC and PC (r = −0.18) in one environment.

### 2.2. Hd1 Allele Types of 293 Korean Rice Cultivars

**Figure 3:**
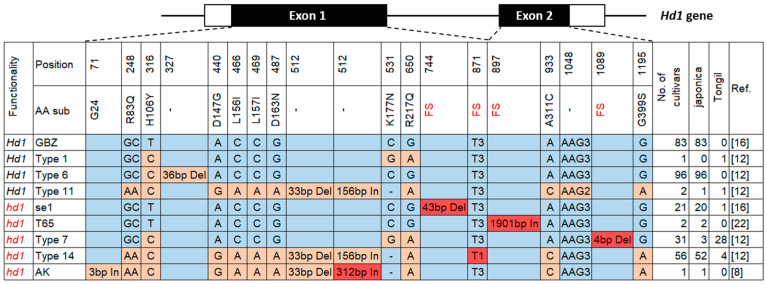
*Hd1* allele types in 293 Korean rice cultivars. *Hd1* and *hd1* indicate functional *Hd1* and nonfunctional *hd1* alleles, respectively. The positions for the nucleotide polymorphisms are according to the *Hd1* gene of the japonica rice cultivar, Ginbouzu (GBZ; GenBank accession AB041840.1). Nucleotide polymorphisms responsible for loss-of-function are highlighted in red. AA—amino acid; FS—frame shift.

To study the allelic distributions of *Hd1* in 293 Korean rice cultivars, we sequenced the protein coding regions of *Hd1* and determined the allele type of each cultivar (Figure 3). This revealed nine different previously reported *Hd1* alleles distributed in the 293 Korean rice cultivars. According to previous reports (Figure 3), four alleles (i.e., GBZ, Type 1, Type 6, and Type 11) were classified as functional *Hd1* alleles, and five alleles (i.e., se1, T65, Type 7, Type 14, and AK) were classified as nonfunctional *hd1* alleles. Out of the 293 Korean rice cultivars, 182 (62.1%) carried functional *Hd1,* while 111 (37.9%) carried nonfunctional *hd1*. Among the 258 japonica cultivars, 180 carried *Hd1* while 78 carried *hd1*. Among the 35 Tongil-type (derived from indica–japonica cross) cultivars, only two carried *Hd1,* while 33 carried *hd1*.

The most frequent functional *Hd1* allele type among the Korean rice cultivars was Type 6 (Nipponbare type), carried by 96 cultivars, followed by the GBZ type (Ginbouzu type), carried by 83 cultivars (Figure 3, Table 1) [12,16]. All of the cultivars carrying Type 6 (*n* = 96) and GBZ type (*n* = 83) *Hd1* were japonica. Type 11 *Hd1* was carried by one japonica cultivar, Sangnambatbyeo, and one Tongil-type cultivar, Nokwoo [12]. Type 1 *Hd1* was carried by one Tongil-type cultivar, Mimyeon [12].

Among the five nonfunctional *hd1* allele types distributed in Korean rice cultivars, Type 14, due to the 2-bp deletion in exon 2, was the most common, carried by 52 japonica and 4 Tongil-type cultivars (Figure 3 and Table 1) [12]. The second most common *hd1* allele was Type 7 due to the 4-bp deletion in exon 2 [12], carried by three japonica and 28 Tongil-type cultivars. The se1 type *hd1* due to the 43-bp deletion in exon 1, which was initially identified in the gamma irradiation mutant HS66 [16], was carried by 20 japonica cultivars and 1 Tongil-type cultivar, Mogyang. The T65 type *hd1*, due to the 1901 bp retrotransposon insertion in exon 2, which was initially identified in the japonica cultivar Taichung 65 [22], was carried by two Korean japonica cultivars, Jungmo1043 and Naepungbyeo 2. The AK type *hd1* due to the 312-bp insertion in exon 1 was reported in the Japanese rice cultivar Akage (AB300058.1) [8], and the same 312-bp insertion was also reported as the nonfunctional hap11 and *hd1*-*3* [13,23]. Only one Korean cultivar, Jinbuolbyeo, carried the AK type *hd1*.

### 2.3. Association of Hd1 Allele Types with Major Agronomic Traits

To study the effects of *Hd1* allele types on DTH, CL, AC, and PC, one-way ANOVAs were conducted for each trait under four different environments, with the nine *Hd1* allele types as a single factor (Table 2). The effect of *Hd1* allele types was highly significant (*p* < 0.0001) for DTH, CL, and PC in all four environments, and explained 51.4–65.8% of the DTH variation, 13.2–24.2% of the CL variation, and 17.0–53.0% of the PC variation. However, the effect of *Hd1* allele types on AC was significant (*p* < 0.05) in only one (Suwon 2018) of the four environments, explaining 5.5% of the phenotypic variation, indicating the small effect of *Hd1* on AC. This observation was consistent with the weak correlations (0.18 < r < 0.23) between DTH and AC (Figure 2), and is likely because AC is mainly determined by the starch-synthesis related genes such as *GBSSI* and *SSIIa* in rice [24].

For mean comparisons of DTH, CL, and PC, which showed significant differences according to *Hd1* allele types in all four environments, additional ANOVAs were conducted with environments as blocks (a random variable) and *Hd1* allele types as a fixed variable (Table 3). On average, cultivars carrying nonfunctional *hd1* alleles headed 71.8 days after transplanting, which was 13.7 days earlier (*p* < 0.0001) than those carrying functional *Hd1* alleles that headed 85.5 days after transplanting. There were significant DTH differences within the *Hd1* and *hd1* allele groups as well, e.g., Type 6 (84.5 days) exhibiting significantly earlier DTH than GBZ (86.9 days) in the functional *Hd1* group, and Type 14 (68.9 days) exhibiting significantly earlier DTH than Type 7 (80.6 days) in the nonfunctional *hd1* group. The difference in CL between cultivars carrying *Hd1* (76.3 cm) and *hd1* (75.3 cm) was significant (*p* < 0.05) but small (Table 3). Among the nine *Hd1* allele types, remarkable differences in CL were observed between Type 11 *Hd1* (101.1 cm) and the AK type *hd1* (58.6 cm), while the CL range of the other seven allele types was 72.0–79.3 cm. Similar to CL, a significant (*p* < 0.0001) but small difference was observed in PC between cultivars carrying *Hd1* (6.1%) and *hd1* (6.7%) (Table 3). Among the nine *Hd1* allele types, Type 7 *hd1* (6.8%) and the AK type *hd1* (7.1%) exhibited a significantly higher PC than the GBZ type *Hd1* (6.0%), while there was no significant difference in PC among the rest of the allele types.

## 3. Discussion

Heading date optimization is an important goal in rice breeding for enhancing land use efficiency by enabling diverse double cropping systems between rice and other crops, as well as ensuring stable yield and grain quality under target environments. Developing early heading rice cultivars is especially important in high latitude and mountainous areas where early heading characteristics are required for rice plants to complete grain filling before the risk of cold winter [3]. Early heading rice cultivars are also useful for avoiding extreme whether events due to climate change and mitigating methane emissions from rice paddies by minimizing the duration of rice cultivation [25,26]. To exploit such advantages, tremendous efforts have been made in Korean rice breeding programs to develop a number of early heading rice cultivars [19,20]. However, the gene mechanisms underlying heading date variation of Korean rice cultivars are unclear. In this study, we determined the allele types of *Hd1*, the major rice heading date gene, in 293 Korean rice cultivars, and investigated their associations with major agronomic traits to provide useful information for rice breeding programs.

A total of nine *Hd1* allele types, four functional and five nonfunctional alleles, were distributed in 293 Korean rice cultivars (258 japonica and 35 Tongil-type derived from indica–japonica crosses). The two most frequent functional *Hd1* alleles, Type 6 (n = 96) and the GBZ type (n = 83), were carried only by japonica cultivars (Table 1). These two alleles were present only in temperate japonica accessions among diverse rice genotypes, as well as those evaluated at IRRI [15], indicating that they are japonica specific alleles. The other two functional allele types, Type 1 and Type 11, were rare in Korean rice cultivars, carried by only one (Mimyeon) and two (Sangnambatbyeo and Nokwoo), respectively.

Among the five nonfunctional *hd1* allele types, the most frequent was Type 14, due to the 2 bp deletion in exon 2, which was carried by 52 japonica and four Tongil-type cultivars. Type 14 *hd1* was initially reported in the indica cultivar Kasalath [16], and five different alleles (Types 13, 14, 15, 16, and17) with the same 2 bp deletion were subsequently identified and classified as nonfunctional *hd1* alleles [12]. This mutation has been identified over 700 rice accessions belonging to different subgroups encompassing japonica, indica, aus, intermediate, and landraces among IRRI’s 3 K rice accessions [18], indicating that it has been present before the indica–japonica divergence and is widely utilized to expand the adaptability of different rice subgroups. The second most frequent *hd1* allele was Type 7, due to the 4 bp deletion in exon 2, which was carried by 28 Tongil-type and three japonica cultivars. This mutation was also frequent among IRRI’s 3 K rice accessions, carried by over 400 accessions, of which 99% were indica [18], indicating that it is likely to have occurred after the indica–japonica divergence and has been popularly utilized in developing Tongil-type cultivars in Korea. The se1 type *hd1* due to the 43 bp deletion in exon 1 has been widely utilized in rice breeding in Japan, China, and Europe [6,8,17], and also for developing early heading Korean rice cultivars, as seen in 20 japonica and 1 Tongil-type cultivars in this study. The T65 type *hd1* due to the 1.9 kb retrotransposon insertion in exon 2 was initially reported in the japonica cultivar Taichung65, and was subsequently identified in rice accessions from China, Cambodia, and Nepal [18,22]. Only two Korean cultivars, Jungmo1043 and Naepungbyeo, carried the T65 type *hd1*. The AK type *hd1* due to the 312 bp insertion in exon 1 is frequently found in rice cultivars developed in the Hokkaido (41°2′–45°3′ N latitude) region of Japan, which is considered as one of the northern limits of rice cultivation [8]. This mutation was carried by only one Korean cultivar, Jinbuolbyeo, which showed the earliest heading date among 293 Korean rice cultivars investigated in this study. Further work is required to determine if the earliness of Jinbuolbyeo is mainly due to the AK type *hd1* or if there are other genetic factors affecting the early heading characteristics of this cultivar.

Nine *Hd1* allele types explained 51.4–65.8% of the DTH variation of 293 Korean rice cultivars under four different environments (Table 2), demonstrating the substantial role of *Hd1* as a single gene determining the heading dates of Korean rice cultivars. On average, 111 cultivars carrying nonfunctional *hd1* headed 14 days earlier than 182 cultivars carrying functional *Hd1*. While many previous studies evaluated the effects of different *Hd1* alleles similarly by classifying them into two categories, functional *Hd1* and nonfunctional *hd1* [12,13,15], our study showed that large DTH variation exists also within each category (Table 3), demonstrating the need for evaluating the effects of each individual allele precisely. Studies using near isogenic lines (NILs) carrying different *Hd1* alleles showed that missense mutations in exon 1 (i.e., N165K and R205Q) induce functional differences in the *Hd1* protein and indicated that *Hd1* sequence variation other than loss-of-function mutations can also affect rice heading dates [7]. Therefore, for fine-tuning heading dates in rice molecular breeding programs, it is important to characterize the function of different alleles within both *Hd1* and *hd1* categories under an isogenic background to precisely evaluate their effects on DTH. In addition, as genes other than *Hd1* also affect rice heading dates, cataloging the allele types of other major rice heading date genes such as *Ghd7*, *DTH8*, and *Hd3a* in different rice cultivars, and evaluating their effects on DTH would facilitate rice breeding programs aimed at precisely controlling heading dates under target environments.

The heading date is known to be associated with a range of agronomic traits affecting yield, such as plant height, panicle number, grain number, and weight, and those affecting eating and cooking quality, such as AC and PC [3,27,28,29,30]. Therefore, to facilitate the use of different *Hd1* alleles in rice molecular breeding, it is important to evaluate the pleiotropic effects of *Hd1* and the effects of different *Hd1* alleles on major agronomic traits. Our study using 293 Korean rice cultivars showed that nine *Hd1* allele types explain 13.2–24.2% of CL variation and 17.0–53.0% of PC variation under four different environments, while explaining only 5.5% of AC variation under only one environment (Table 2). In addition, the differences in CL and PC between the cultivars carrying functional *Hd1* and nonfunctional *hd1* were significant but small (Table 3). Our results indicate that it would be possible to use different *Hd1* alleles to modulate DTH, while affecting CL, AC, and PC to a mild extent. The replacement of the GBZ type (aka Haplotype 8) *Hd1* carried by the japonica rice cultivar Chunjiang06 with Type 7 (aka Haplotype 16) resulted in a yield improvement without affecting the grain quality traits, such as gel consistency and gelatinization temperature [17], supporting that the use of different *Hd1* alleles can optimize DTH effectively without negatively influencing other agronomic traits. As rice cultivars with different genetic background used in this study provide a limited capability for precisely evaluating the effects of different *Hd1* alleles on agronomic traits, further studies using isogenic lines would be necessary to generate more precise information that can be utilized in rice breeding programs for selecting optimal *Hd1* alleles, meeting different breeding objectives.

In conclusion, we cataloged the allele types of *Hd1* distributed in Korean elite rice cultivars and evaluated their associations with DTH, CL, AC, and PC under four different field environments. While the *Hd1* allele types explained a large proportion (51.4–65.8%) of DTH variation, their effects on CL, AC, and PC were limited. Our study and further work to characterize the precise allelic effects of *Hd1* and other heading date genes will advance rice molecular breeding tools for fine-tuning DTH and other important agronomic traits.

## 4. Materials and Methods

### 4.1. Plant Materials and Phenotyping

A total of 293 Korean rice cultivars released by the National Institute of Crop Science (NICS), of the Rural Development Administration (RDA) of South Korea between 1979 and 2017 were used in this study. The list of the cultivars initially described in Lee et al. [20] contains 297 cultivars, but we excluded four with potential seed contamination in this study and used only 293 cultivars.

The rice plants were cultivated at the experimental field stations of NICS located in Wanju (35°84′ N 127°05′ E) and Suwon (37°27′ N 126°99′ E) in 2018 and 2019. In 2018, the plants were cultivated in optimum planting conditions with sowing and transplanting dates of 9 May and 1 June in Wanju, and 25 April and 25 May in Suwon, respectively. In 2019, the plants were cultivated in early planting conditions with the sowing and transplanting dates of 10 April and 9 May in Wanju, and 10 April and 10 May in Suwon, respectively. Each cultivar was transplanted in four 4.5 m rows with the individual plants spaced by 15 cm, and the rows were spaced by 30 cm. The plants were grown and managed according to the standard rice cultivation methods of NICS, RDA [31].

Days to heading (DTH) were determined by counting the days from transplanting to heading, when 40% of the plants of each cultivar exhibited emerged panicles. Culm length (CL) was determined by measuring the length from the ground to the panicle node of the main culm from 10 random plants, and averaging them to represent each cultivar. Upon maturity, the rice grains were harvested, dried at 15% grain moisture content naturally under shaded condition, dehulled with a roller husking machine (SY88-TH; Ssangyong Ltd., Incheon, Korea), and polished with a laboratory polishing machine (MC-90A; Toyo Co., Wakayama, Japan) as previously described [32]. The amylose content (AC) was evaluated according to Juliano [33] using the iodine colorimetric method with a UV/visible spectrophotometer (Evolution 600; Thermo Fisher Scientific, Waltham, MA, USA). Protein content (PC) was evaluated using the Micro Kjeldahl method according to AOAC [34] with automated Kjeldahl analyzers (Foss Digester 2020 and Foss Kjeltec 2400; Foss Tecator, Huddinge, Sweden).

### 4.2. Hd1 Genotyping

Leaf tissues were collected from 3-week-old rice seedlings and genomic DNA was extracted using the CTAB method [35]. To determine the *Hd1* allele types of the 293 Korean rice cultivars by sequencing the protein coding regions of *Hd1*, each of the two exons of *Hd1* was amplified by a pair of primers (*Hd1*_exon1_F and *Hd1*_exon1_R for the first exon, and *Hd1*_P4_F and *Hd1*_P4_R for the second exon) described in Table 4. For the first exon, three additional primers (*Hd1*_P1_R, *Hd1*_exon1_R1, and *Hd1*_P2_F) were used as the inner primers for sequencing. For sequencing the 312 bp and 1901 bp insertions identified in the first exon of several cultivars, two (*Hd1*_P1_F and *Hd1*_P2_R) and four (*Hd1*_exon1_F1, *Hd1*_exon1_F2, *Hd1*_exon1_R2, and *Hd1*_exon1_R3) additional primers were used as the inner primers. For the second exon, the primers used for PCR were also used for sequencing. The Sanger sequencing reactions were conducted using the Capillary Electrophoresis Sequencing (CES) service at Macrogen, South Korea (https://dna.macrogen.com, accessed on 13 October 2021).

### 4.3. Statistical Analysis

All of the statistical analyses and data visualization were conducted using R (The R Project for Statistical Computing, version 4.1.1). Boxplots with data points were drawn using the packages “ggplot2”, “tidyverse”, “hrbrthemes”, and “viridis”. Correlation analyses and their visualization were conducted using the packages “Hmisc” and “corrplot”. Analyses of variance (ANOVA) were conducted using the package “agricolae”.

## Figures and Tables

**Table 1:** *Hd1* allele types of 293 Korean rice cultivars.

Type ^z^	Cultivars ^y^
GBZ (*Hd1*)	Anbaek, Anmi, Baegjinju, Baegjinju1ho, Boramchan, Borami, Cheongan, Cheongdam, Cheonghaejinmi, Cheongun, Chindeul, Chinnong, Dacheong, Daejinbyeo, Daesanbyeo, Dami, Deuraechan, Donghaejinmi, Dongjin2, Dongjinbyeo, Gancheokbyeo, Gangbaek, Gangchan, Geonyang2, Geuman, Geunnun, Gihobyeo, Goami3, Goami4, Haepum, Hanam, Hanmauem, Heugkwang, Heugseol, Hopum, Huimangchan, Hwaan, Hwanggeumnodeul, Hwangkeumnuri, Hwarang, Hwaseongbyeo, Hyeonpum, Ilmibyeo, Ilpumbyeo, Jannganbyeo, Jinbaek, Jinpum, Jinsumi, Jonong, Joryeongbyeo, Junam, Keumobyeo1, Keunpum, Kuemobyeo, Mananbyeo, Manbaek, Migwang, Miho, Misiru, MY298BB, MY299BK, Nunbora, Onnuri, Saeilmi, Saeilpum, Saenuri, Saesin, Sampyeong, Seoan1ho, Seoanbyeo, Seolgaeng, Seomyeong, Shinbaeg, Sindongjin, Sinjinbaek, Sodami, Suan, Sukwang, Yangjobyeo, Yechan, Yeongan, Younghojinmi, and Youngjin
Type 1 (*Hd1*)	Mimyeon
Type 6 (*Hd1*)	Aranghyangchalbyeo, Aromi, Baegokchal, Baegseolchal, Bodrami, Boramchal, Boseogchal, Boseogheugchal, Cheonga, Cheongcheongjinmi, Cheongho, Cheonghyangheukmi, Cheongnam, Cheongpum, Chilbo, Dabo, Daeanbyeo, Daebo, Daecheongbyeo, Daepyeong, Daeripbyeo1, Danmi, Dodamssal, Donganbyeo, Dongbo, Dongjin1ho, Dongjinchalbyeo, Geonganghongmi, Geonyangmi, Goami, Gopum, Gyehwabyeo, Haechanmulgyeol, Haepyeong, Haepyeongchal, Haiami, Hangaru, Heaoreumi, Heughyang, Heugjinmi, Heugnambyeo, Heugsujeong, Hoanbyeo, Hojin, HONGJINJU, Honong, Hopyung, Hwabong, Hwajinbyeo, Hwajungbyeo, Hwanambyeo, Hwasambyeo, Hwaseonchalbyeo, Hwasinbyeo, Hwayeongbyeo, Hyangnambyeo, Jeogjinju2, Jinbo, Jinkwang, Juanbyeo, Jungmo1006, Jungmo1032, Jungmo1034, Jungsaenggold, Malgeumi, Mangeumbyeo, Manjong, Manmi, Manpung, Manwol, Mihyangbyeo, Mipum, Misomi, Nampyeongbyeo, Palgongbyeo, Pungmi, Pungmi1, Saechilbo, Saegoami, Saegyewha, Samdeog, Samkwang, Samkwang1ho, Sangbo, Seolhyangchal, Seonpum, Seopyeong, Sinbo, Sinseonchalbyeo, Sobi, Sujin, Surabyeo, Suryeojinmi, Tamjinbyeo, Yeongdeogbyeo, and Youngbo
Type 11 (*Hd1*)	Sangnambatbyeo and Nokwoo
se1 (*hd1*)	Asemi, Baegilmi, Danpyeng, Hanseol, Heukjinjubyeo, Hwangkeumbora, Hwawang, Jeogjinju, Jinbuchalbyeo, Jinmibyeo, Jinseolchal, Jogwang, Jopum, Josaengheugchal, Joun, Jungsan, Manchu, Manho, Obongbyeo, Ondami, and Mogyang
T65 (*hd1*)	Jungmo1043 and Naepungbyeo
Type 7 (*hd1*)	CW92MR, Keumo3, Nonganbyeo, Andabyeo, Areumbyeo, Cheongcheongbyeo, Cheongwoo, Dasan1ho, Dasan2, Dasanbyeo, Geumgang1, Hanareum, Hanareum2, Hanareum3ho, Hanareum4, Hanareumchal, Hangangchal1, Hangangchalbyeo, Hyangmibyeo1, Jangseongbyeo, Jungwonbyeo, Keunseom, Namcheonbyeo, Nampungbyeo, Palbangmi, Saemimyeon, Samgangbyeo, Segyejinmi, Singil, Taebaekbyeo, and Yongmunbyeo
Type 14 (*hd1*)	Asemi1ho, Boseog, Cheongbaekchal, Dunnaebyeo, Geumobyeo, Geumyoung, Goun, Gurubyeo, Haedamssal, Haedeul, Handeul, Jinbubyeo, Jinhan, Jinok, Joami, Joan, Joeunheukmi, Joil, Jopyeong, Junghwabyeo, Jungmo1024ho, Manna, Namil, Namwonbyeo, Nunkeunheugchal, Nunkeunheugchal1ho, Odae1ho, Odaebyeo, Pyeongwon, Saeodae, Saesangju, Samcheonbyeo, Sandeuljinmi, Sangjubyeo, Sangjuchalbyeo, Sanhomi, Seolbaek, Seolemi, Seongsan, Shinpyeong, Sinunbong1, Sinunbongbyeo, Sobaegbyeo, Taebong, Unbaekchal, Unbongbyeo, Undoobyeo, Unilchal, Unjangbyeo, Unkwang, Unmi, Wolbaek, Miwoo, Mogwoo, Nokyang, and Yeongwoo
AK	Jinbuolbyeo

^z^ *Hd1* and *hd1* in parentheses indicate functional and nonfunctional alleles, respectively. ^y^ Tongil-type cultivars derived from indica × japonica crosses are underlined.

**Table 2:** Effects of *Hd1* allele types on major agronomic traits of 293 Korean rice cultivars.

ENV ^z^	DTH		CL		AC		PC	
	F-Value	PVE (%)	F-Value	PVE (%)	F-Value	PVE (%)	F-Value	PVE (%)
WJ 2018	37.6 ****	51.4	5.4 ****	13.2	0.9 ^NS^	-	40.0 ****	53.0
WJ 2019	58.3 ****	62.2	6.2 ****	14.8	1.4 ^NS^	-	7.2 ****	17.0
SW 2018	67.3 ****	65.5	11.4 ****	24.2	2.1 *	5.5	15.1 ****	29.8
SW 2019	68.2 ****	65.8	6.9 ****	16.2	1.4 ^NS^	-	33.9 ****	42.2

^z^ Four environments composed of two locations, Wanju (WJ) and Suwon (SW), and two consecutive years, 2018 and 2019. One-way ANOVAs were conducted in each environment for each trait, with nine *Hd1* allele types as a single factor. DTH—days to heading; CL—culm length; AC—amylose content; PC—protein content; PVE—phenotypic variance explained by the *Hd1* allele types. **** *p* < 0.0001, * *p* < 0.05, ^NS^ not significant.

**Table 3:** Mean comparisons of days to heading (DTH), culm length (CL), and protein content (PC) according to *Hd1* allele types in 293 Korean rice cultivars.

*Hd1* Allele ^z^	No. of	DTH	CL	PC
	Cultivars	(Days)	(cm)	(%)
GBZ (*Hd1*)	83	86.9 ± 8.42 a	75.7 ± 8.36 c	6.0 ± 0.59 b
Type 1 (*Hd1*)	1	76.9 ± 9.13 cd	79.3 ± 7.16 bc	6.8 ± 0.49 ab
Type 6 (*Hd1*)	96	84.5 ± 8.66 b	76.3 ± 8.82 c	6.2 ± 0.63 ab
Type 11 (*Hd1*)	2	81.2 ± 15.29 bc	101.1 ± 21.88 a	6.5 ± 0.67 ab
se1 (*hd1*)	21	67.8 ± 9.18 d	73.8 ± 8.39 c	6.7 ± 1.05 ab
T65 (*hd1*)	2	71.0 ± 6.72 d	72.0 ± 8.08 cd	6.7 ± 0.78 ab
Type 7 (*hd1*)	31	80.6 ± 9.17 c	79.3 ± 6.82 b	6.8 ± 0.71 a
Type 14 (*hd1*)	56	68.9 ± 10.01 d	74.0 ± 8.30 c	6.7 ± 0.95 ab
AK (*hd1*)	1	51.9 ± 5.72 e	58.6 ± 3.41 d	7.1 ± 1.37 a
*Hd1*	182	85.5 ± 8.74	76.3 ± 9.19	6.1 ± 0.62
*hd1*	111	71.8 ± 11.10 ****	75.3 ± 8.39 *	6.7 ± 0.91 ****

^z^ *Hd1* and *hd1* in parentheses indicate functional and nonfunctional alleles, respectively. DTH—days to heading, CL—culm length; PC—protein content. The values for each trait are mean ± standard deviation from four different environments. Different lowercase letters next to the trait values indicate significant difference according to Tukey’s multiple comparisons at *p* < 0.05. **** *p* < 0.0001, * *p* < 0.05.

**Table 4:** PCR and sequencing primers used for *Hd1* genotyping.

Region	Usage	Primer Name	Sequence	Reference
Exon1	PCR	*Hd1*_exon1_F	CCAAGTGTCAATCGCTGGAT	this study
		*Hd1*_exon1_R	AAGTAGTCAACTGGTCTGCC	this study
	Sequencing	*Hd1*_P1_R	CGGTTGTCGTAGTACGAATTGTAC	[15]
		*Hd1*_exon1_R1	ATCTTGGTTGTTTTCGATGCG	this study
		*Hd1*_P2_F	ACGAGGAGGTGGACTCTTG	[15]
	312 bp insertion ^a^	*Hd1*_P1_F	TTCCCCTCCCTAGCTCCTTCCAA	[15]
		*Hd1*_P2_R	ATCGGTTCCATTTAATCAGCCT	[15]
	1.9 kb insertion ^b^	*Hd1*_exon1_F1	ATCAGTGGGCCTTGGTTATG	this study
		*Hd1*_exon1_F2	GAAACAGCGACAATGACGAC	this study
		*Hd1*_exon1_R2	GCATCTTCTTCACCGAGTCC	this study
		*Hd1*_exon1_R3	ATCACCCCCTAAACCTGACC	this study
Exon2	PCR and sequencing	*Hd1*_P4_F	GAAAGACCTCATGAAAAGTAGG	[15]
		*Hd1*_P4_R	GCTATCCGGAAATTACAAAGCA	[15]

^a^ Additional primers used for sequencing the 312 bp insertion identified in Jinbuolbyeo. ^b^ Additional primers used for sequencing the 1.9 kb insertion identified in Jungmo1043 and Naepungbyeo.

## Data Availability

The data generated in this study are available from the corresponding author upon reasonable request.
